# Supplementary material for: How does the institutional environment improve the entrepreneurial quality of returnees? A configuration analysis based on a complex system view
Source: PLoS One. 2025 Jul 1;20(7):e0322863. doi: 10.1371/journal.pone.0322863 (PMC12212558; doi:10.1371/journal.pone.0322863)
Supplement: Attachment — Submitted filename: Comments.docx [file pone.0322863.s002.docx]

**How does the institutional environment improve the entrepreneurial quality of returnees?A configuration analysis based on a complex system view**

I express profound gratitude to review the article.This study analyzes the complex relationship between institutional configurations and the quality of returnee entrepreneurship in China. The research samples include 28 regions. The subsequent issues pertain to the article are as follows.

**Introduction**

- Add research objectives and research questions in the introduction section in bullets.

**Theoretical Background and Literature Review**

- Add theoretical background. Add theories related to the topic.
- Add research Hypothesis in bullets,
- Before starting methodology there is a typing error” **Fig 1. This is the Fig 1 Title.**This si the Fig 1 Legend”.

**Empirical Results Discussion and Implications**

- Add the discussion part at the end of the empirical results. In which provide overall discussion regarding results and its impacts and implications.
