# Supplementary material for: How does the institutional environment improve the entrepreneurial quality of returnees? A configuration analysis based on a complex system view
Source: PLoS One. 2025 Jul 1;20(7):e0322863. doi: 10.1371/journal.pone.0322863 (PMC12212558; doi:10.1371/journal.pone.0322863)
Supplement: Attachment — Submitted filename: Recommendation of Article.docx [file pone.0322863.s004.docx]

**Recommendation of Article:**

**How does the institutional environment improve the entrepreneurial quality of**

**returnees? A configuration analysis based on a complex system view**

- In-text citations problems persist like name of author and brackets with number
- Fiss (2011)[66]
- Formatting problems persist in headings and proper numbering is missing
- Important research papers that considered to be base papers are missing e.g.
- Returnee entrepreneurs and the institutional environment: case study insights from ChinaJanuary 2019 [International Journal of Emerging Markets](https://www.researchgate.net/journal/International-Journal-of-Emerging-Markets-1746-8809?_tp=eyJjb250ZXh0Ijp7ImZpcnN0UGFnZSI6InB1YmxpY2F0aW9uIiwicGFnZSI6InB1YmxpY2F0aW9uIn19) 14(1):207-230 DOI:[10.1108/IJoEM-11-2017-0504](http://dx.doi.org/10.1108/IJoEM-11-2017-0504)
- Bomzon SD, Mukherjee S, Murti AB. The Process of Returnee Entrepreneurship: From Firm Creation to Contribution. InAcademy of Management Proceedings 2024 (Vol. 2024, No. 1, p. 18083). Valhalla, NY 10595: Academy of Management.
- Repetition of concepts and words in whole article. Please give a proper read and omit them.
