# Supplementary material for: How does the institutional environment improve the entrepreneurial quality of returnees? A configuration analysis based on a complex system view
Source: PLoS One. 2025 Jul 1;20(7):e0322863. doi: 10.1371/journal.pone.0322863 (PMC12212558; doi:10.1371/journal.pone.0322863)
Supplement: Attachment — Submitted filename: 2nd article Review.docx [file pone.0322863.s005.docx]

**Review of How does the institutional environment improve the entrepreneurial quality of returnees?**

**Review 1:** Although the topic is very interesting but

- Research Objectives/Questions needs to be highlighted properly. Research gap need to be addressed in the light of theory.

**Review 2:**

- Add research hypotheses and authors presenting model without any support of hypotheses.
- Connect theory with hypotheses. H1, H2, and H3 and discuss hypotheses in the light of the theory.
- Furthermore, provide hypotheses as you mentioned in Configuration Analysis for period-1 H1, (H1a), (H1b), and (H1c). Similarly in period 2 and 3^.^

**Review 3:**

- You normalize the data at first stage but how and which method you use to normalize the data. Please provide details. Also furnish detail about calibration method.
