# Supplementary material for: How does the institutional environment improve the entrepreneurial quality of returnees? A configuration analysis based on a complex system view
Source: PLoS One. 2025 Jul 1;20(7):e0322863. doi: 10.1371/journal.pone.0322863 (PMC12212558; doi:10.1371/journal.pone.0322863)
Supplement: Attachment — Submitted filename: Response to Reviewers.docx [file pone.0322863.s007.docx]

**Manuscript Number:** PONE-D-24-40215

**Title:** How does the institutional environment improve the entrepreneurial quality of returnees? A configuration analysis based on a complex system view

**Corresponding Author Email:** [yht@hqu.edu.cn](mailto:yht@hqu.edu.cn)

**Response to Reviewers**

Dear Editor and Reviewers:

We are grateful for the editor and all reviewers for the constructive remarks and valuable suggestions, which have significantly improved the quality of our manuscript it. We have taken the reviewers’ suggestions into deep consideration and revised our work carefully regarding those.

Below the reviewers' comments, we have made direct responses and presented revised content (highlighted in blue) point by point.

**Reviewer 2**

***Question1:*** *Although the topic is very interesting but Research Objectives/Questions needs to be highlighted properly. Research gap need to be addressed in the light of theory.*

**Response:** Thank you very much for your meticulous review, esteemed reviewer! As you have pointed out, it is crucial to highlight the research objectives/questions and theory gap clearly. We acknowledge that our expression in this section was not as definitive as it could have been. In response to your feedback, we have further refined the presentation of the research questions/objectives and theory gap. We sincerely hope that our improvements will meet with your approval and enhance the clarity of the introduction. Thank you once again for your valuable and insightful feedback!

**Modified in Manuscript:**

On page 1 in blue. “Returnee entrepreneurs have the potential to enrich emerging market economies by introducing advanced technologies, substantial capital, and a diverse cultural perspective [1, 2]. They are gradually becoming a significant driving force for the rapid financial development of emerging economies. With ongoing improvements in the institutional environment of these economies, returnees are increasingly motivated to establish businesses in their home countries [3, 4]. However, companies founded by returnees often encounter challenges stemming from their external status and limited contextual relevance. These obstacles can slow their development compared to domestic companies, impeding their ability to achieve high-quality entrepreneurship [5]. The role of the institutional environment is crucial for entrepreneurial development and economic growth [6, 7] , provided that high-quality entrepreneurial activities are the core engine of high-quality economic development [8]. Notably, there is a significant geographic concentration of high-quality returnee entrepreneurial activities in China, as evidenced by the "2021 China Overseas Returnees' Innovation and Entrepreneurship Ranking", where 70% of the top 20 companies are located in first-tier cities. This underscores the urgency of optimizing the institutional environment to narrow regional development disparities and enhance the quality of returnee entrepreneurship. Therefore, the research question of this paper is: How does the institutional environment in emerging economies promote the entrepreneurial quality of returnees?”

On page 2 in blue. “First of all, Current research on institutional influences in entrepreneurship remains constrained by disciplinary silos, predominantly relying on sociological institutional theory [13]. While existing studies effectively elucidate how institutional legitimacy and resource allocation mechanisms shape entrepreneurial outcomes, two critical theoretical limitations persist. On the one hand, the exclusive focus on normative-cognitive institutional forces overlooks transaction costs in entrepreneurial decision-making. On the other hand, the lack of cross-paradigm dialogue between sociological and economic institutional theories hinders a comprehensive understanding of institutional complexity [13, 16]. This study innovatively combines institutional theories from both sociology and economic theory. It is grounded in sociological institutional theory, highlighting key drivers of high-quality returnee entrepreneurship, including organizational legitimacy and the alleviation of institutional barriers [10, 14, 15]. It also integrates the economic transaction cost theory to show how the institutional environment improves entrepreneurial quality. By reducing information asymmetry and lowering transaction costs, it facilitates the flow of technological innovation resources [16]. This integrated perspective provides a more nuanced analytical framework for explaining variations in entrepreneurial activities across regions.”

***Question2:*** *(1)Add research hypotheses and authors presenting model without any support of hypotheses.*

*(2)Connect theory with hypotheses. H1, H2, and H3 and discuss hypotheses in the light of the theory.*

*(3)Furthermore, provide hypotheses as you mentioned in Configuration Analysis for period-1 H1, (H1a), (H1b), and (H1c). Similarly in period 2 and 3.*

**Response:** Thank you for your in-depth review and valuable suggestions. We truly appreciate your emphasis on research rigor in suggesting to "add research hypotheses (H1, H2, H3, etc.) and connect theory with hypotheses." We fully understand the central role of hypothesis testing in traditional empirical research. However, based on the methodological characteristics of QCA (Qualitative Comparative Analysis) and our research design, we would like to explain in detail why QCA studies typically do not rely on traditional hypotheses.

Qualitative Comparative Analysis is a set-theory and Boolean-algebra-based method that explains complex social phenomena by identifying combinations of conditions ("configurations"). Its core idea is "causal complexity," where outcomes result from the joint action of multiple conditions rather than a single variable (Ragin, 2008). QCA emphasizes the following principles: ① Equifinality: The same outcome can be achieved through different combinations of conditions. ② Asymmetry: The combinations leading to the presence and absence of an outcome may differ. ③ Necessary and sufficient conditions: Analyze the necessity or sufficiency of conditions for an outcome using calibrated data (like fuzzy sets). QCA is suitable for medium-to-small-sample studies (N = 10 - 100) and is often used for exploratory (inductive) research to discover theoretical patterns through case comparison. It is not just a research method but a methodology. Based on the core ideas and principles of QCA methodology, research hypotheses are not required in papers applying this methodology for the following reasons:

First, the essential conflict between QCA's core value and hypothesis testing logic. Traditional hypothesis testing (e.g., regression analysis) assumes variables independently affect outcomes (e.g., H1: X→Y). In contrast, QCA identifies "causal recipes" (e.g., A + B + ∼C→Y), where conditions interact, substitute, or negate each other (Fiss, 2011). This combinational logic cannot be simplified into single hypotheses.

Second, causal complexity goes beyond linear assumptions. QCA deals with nonlinear relationships like "threshold effects" and "asymmetric paths." For example, "the combinations leading to high performance may differ from those leading to low performance" (Misangyi et al., 2017). Forcing linear hypotheses (e.g., A positively affects Y) may oversimplify phenomena and ignore dynamic dependencies between conditions.

Third, QCA is more for theory building than theory verification. It is often used in exploratory research to generate new theories through case comparison (e.g., discovering unrecognized success configurations). "QCA's counterfactual analysis supports theoretical innovation rather than verifying existing propositions" (Schneider & Wagemann, 2012). For instance, researchers might find that "firms with low resources achieve high growth through flexible strategies," a finding that cannot be derived from pre - specified hypotheses.

Fourth, methodological differences. QCA requires researchers to "return to cases" and interpret configurations' rationality using case knowledge (Ragin, 2008). For example, when analyzing multi - temporal data, researchers need to incorporate time dynamics (e.g., trajectory analysis as proposed by Pagliarin & Gerrits, 2020) rather than rely on assumption - driven statistical tests. Including hypothesis testing in the literature review might lead to "method-theory disconnection" (Park et al., 2020).

Moreover, most QCA papers in top journals do not pre-specify research hypotheses, such as Leppänen et al. (2021) in Academy of Management Journal and Howell et al. (2022) in Organization Science.

In summary, considering QCA methodology's characteristics and the prevalent writing patterns in top-journal QCA papers, we have decided not to add a research hypothesis section. We hope our explanation is understandable to you. Thank you!

***Question3:*** *You normalize the data at first stage but how and which method you use to normalize the data. Please provide details. Also furnish detail about calibration method.*

**Response:** Thank you very much for the reviewer's thorough evaluation! We fully appreciate the importance of clearly describing data normalization and calibration methods. In response to your suggestions, we will elaborate on our data preprocessing steps and rationale. We've also enhanced the calibration section, specifying the software, function, and method used in the calibration process. We hope that our enhancements will meet with your approval. Thank you!

**Modified in Manuscript:**

On page 10-11 in blue. “To effectively eliminate the impact of original indicators' dimensions during the measurement phase, we first normalized the raw data. In this study, normalization was performed using SPSS software via the Min-Max normalization method, which standardizes data of varying dimensions by mapping it to the [0, 1] interval. Compared to Z-score normalization (which may yield negative values), the [0, 1] interval better aligns with the set - membership logic of QCA. The formula is:

$$X_{normal=\frac{X-X_{\min}}{X_{\max}-X_{\min}}}$$

where X is the raw data, $X_{\min}$ and $X_{\max}$ are the minimum and maximum values of the dataset, and $X_{\mathrm{normal}}$ is the normalized value. Descriptive statistics of the normalized data are presented in Table 1. Measurement indicators for the six institutional environment factors are as follows:”

On page 13 in blue. “Calibration, a process of assigning set - membership scores to cases, adopts the direct method using the calibrate function in fsQCA software. Drawing on prior research [68], the three calibration points for each variable are set at the upper quartile (full membership threshold), median (crossover point threshold), and lower quartile (full non-membership threshold) of the sample data. To prevent ambiguous 0.5 membership scores that hinder case classification and analysis, a 0.001 upward adjustment is made to scores below 1. The calibration results are shown in Table 1.”

**Editor**

***Question1:*** *In-text citations problems persist like name of author and brackets with number*

*Fiss (2011)[66]*

**Response:** Thank you for the in-depth review. We apologize for the oversight in the manuscript. We've carefully reread and revised the incorrect literature citations. You can see the specific revisions in the modified manuscript. We hope our revisions meet your approval. Thank you again!

***Question2:*** *Formatting problems persist in headings and proper numbering is missing*

**Response:** Thank you for your reminder. We have carefully rechecked the numbering of headings in the manuscript and made appropriate adjustments where necessary. You can see the specific revisions in the modified manuscript. We hope our revisions meet your approval. Thank you again!

***Question3:*** *Important research papers that considered to be base papers are missing e.g.*

- *Returnee entrepreneurs and the institutional environment: case study insights from ChinaJanuary 2019 International Journal of Emerging Markets 14(1):207-230 DOI:10.1108/IJoEM-11-2017-0504*
- *Bomzon SD, Mukherjee S, Murti AB. The Process of Returnee Entrepreneurship: From Firm Creation to Contribution. InAcademy of Management Proceedings 2024 (Vol. 2024, No. 1, p. 18083). Valhalla, NY 10595: Academy of Management.*

**Response:** Thank you for your important suggestion regarding the literature citations. As you pointed out, we had overlooked two key studies on returnee entrepreneurship. We have now added citations for these two papers in the manuscript. You can see the specific revisions in the modified manuscript. Thank you very much!

***Question4:*** *Repetition of concepts and words in whole article. Please give a proper read and omit them.*

**Response:** Thank you very much for your careful review. We acknowledge the redundancy issues in the manuscript. We've carefully proofread the full text and addressed the redundancies by replacing repeated terms, standardizing abbreviations, and simplifying sentences. You can see the specific revisions in the modified manuscript. We hope these improvements meet your approval. Thanks again!

**We present our deepest gratitude for all the comments and suggestions. We truly hope that our refined manuscript would meet your requirements and satisfactions. We are looking forward to the official publication of our work.**
